# Prognostic Markers and Trials in Penile Cancer

**DOI:** 10.5152/tud.2023.22225

**Published:** 2023-05-01

**Authors:** Naufal Naushad, Abdalla A. Deb, Ayman A. Agag, Hosam A. Serag, Vijay K. Sangar

**Affiliations:** 1 Department of Urology, Christie NHS Foundation Trust, Manchester, UK; 2 Department of Urology, James Cook University Hospital, Middlesbrough, UK; 3 Department of Urology, Frimley Park Hospital, Frimley, UK; 4 Department of Urology, University Hospitals Birmingham, Birmingham, UK

**Keywords:** Lymphatic metastasis, penile neoplasms, prognosis, squamous cell carcinoma-related antigen, survival

## Abstract

New tumor biomarkers open the potential for designing personalized therapy for penile squamous cell carcinoma. Despite the initial promising results of some biomarkers, controversy remains due to contradictory studies. Further robust research work is required before incorporating biomarkers in the personalized management of penile cancer. This narrative review aims to highlight some of the most commonly and recently investigated biomarkers of penile cancer and to summarize the ongoing registered clinical trials for the management of penile cancer patients.

**Main Points**

- New tumor biomarkers open the potential for designing personalized therapy for penile squamous cell carcinoma.
- Robust research work is required before incorporating biomarkers in the personalized management of penile cancer.
- This review highlights the recently investigated biomarkers of penile cancer and summarizes the ongoing clinical trials for its management.

## Introduction

The incidence of penile cancer (PC) is rare,^1^ with approximately 26 000 new cases diagnosed annually worldwide. The incidence rate varies depending on the geographic location, with higher incidence rates in some regions of South America, Asia, and Africa than in Western Europe and North America.^2^ A meta-analysis reported the age-standardized incidence of PC (expressed as cases per 100 000 person-years) to be highest in Latin America (1.40 cases), followed by Africa (0.99 cases), North America (0.91 cases), Europe (0.90 cases), then Asia (0.44 cases), while the least incidence was reported in Oceania (0.42 cases).^3^ Studies have demonstrated the highest rate in Brazil where the incidence ranged between 2.9 and 6.8 cases per 100 000.^4,5^ The second-highest rate of PC is reported in Uganda, where 3-4 new cases per 100 000 men are diagnosed annually.^4,6^

Unfortunately, the clinical presentations of invasive PC vary, leading to delayed diagnosis, with poor patient survival in some cases.^7^ One of the most significant prognostic factors in PC is the presence of lymph node (LN) metastases.^8^ Pelvic LN metastases are associated with very low 3- and 5-year survival rates, ranging from 9 to 40.^9-12^ Table 1 demonstrates the reported survival rates according to LN involvement.

Inguinal LN dissection (ILND) is indicated if positive inguinal LNs are detected to limit the spread of the disease. Nevertheless, approximately one-quarter of clinically node-negative patients have microscopic LN metastases^13^ that are not feasibly detected using conventional imaging techniques^14^ but can be detected using sentinel node biopsy techniques based on protocols related to the stage of the disease.^15^ In some regions, prophylactic ILND in cN0 patients is undertaken, resulting in the unnecessary risk of procedure morbidities such as wound breakdown, lymphoceles, and lymphedema.^8,16^ There is no current reliable prognostic biomarker which can be used in primary cancers to predict LN metastasis.

Investigating the association between different biomarkers and relevant variables in PC patients improves our understanding of the pathways of tumor development and progression. This, in turn, has provided new insights into identifying new therapeutic targets.^17^ However, to date, no biomarker has reliably provided an aid to diagnosis which is utilized in mainstream practice.

This narrative review aims to highlight some of the most commonly and recently investigated prognostic biomarkers for nodal disease and survival in PC and to summarize the ongoing registered clinical trials for the management of PC patients.

## Methods

**Figure 1.**
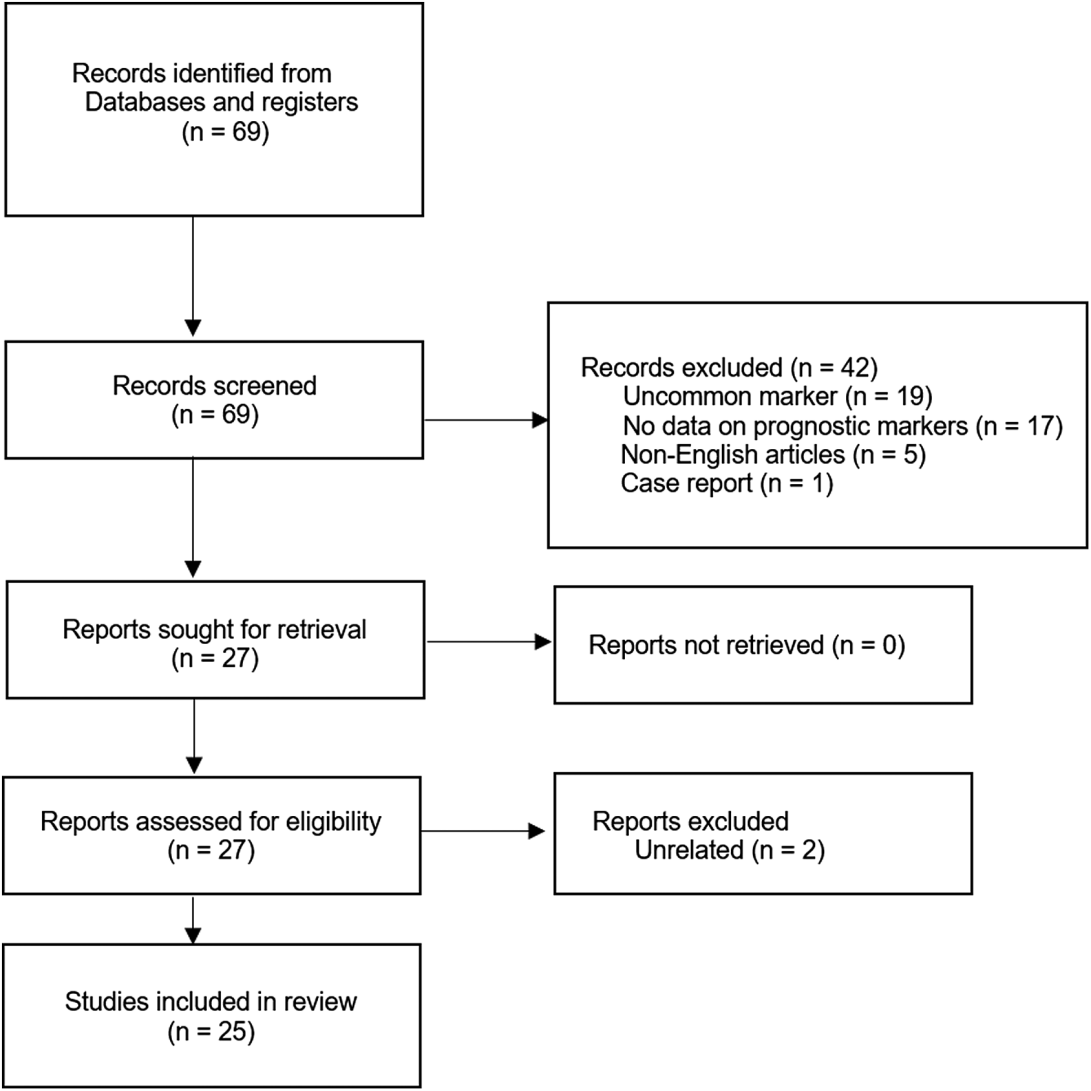
Flow chart of the search results and article selection.

For this narrative review, a literature search was conducted on the databases of PubMed/MEDLINE, Cochrane library, and ClinicalTrials.gov. The search used the keywords “Penile cancer,” “lymph node metastasis,” “survival,” and “biomarkers” to identify relevant articles. The search was limited to English-published articles. The search yielded 69 results, out of which 25 were used. As a large number of biomarkers were evaluated in the retrieved studies, the review focused on the most commonly evaluated or discussed biomarkers. The studies were included if they assessed the prediction of LN metastasis, overall survival (OS), or disease-specific survival (DSS) in patients with PC. In addition, articles that were case reports or did not include data on the prognostic value of the biomarker for lymphatic metastasis or survival were excluded (Figure 1). The reference lists of the retrieved articles were searched for further related studies and previous reviews.

### Penile Cancer Markers

#### Squamous Cell Carcinoma Antigen

The squamous cell carcinoma antigen (SCCAg) is a tumor-associated glycoprotein. Seven studies^18-24^ assessed the potential association between elevated serum levels of SCCAg and LN metastasis in penile squamous cell carcinoma (SCC) patients. A statistically significant association with nodal disease was reported by 4 studies: 2 in univariate analysis^19,22^ and 2 studies in multivariate analysis.^18,20^ Zhu et al^22^ reported that at a cut-off level > 1500 ng/L, the sensitivity and specificity of SCCAg were 34.3% and 89.3%, respectively, for predicting LN metastasis (*P* = .005). However, they found that SCCAg poorly predicted occult inguinal metastasis in clinically node-negative patients (Table 2).

Two studies^19,23^ examined the association between elevated SCCAg levels and survival in patients with penile SCC after surgery (Table 2). Li et al^19^ found that DSS was significantly lower by 28% in patients with elevated SCCAg levels compared to those with normal levels in univariate analysis, but the association was not significant in multivariate analysis (hazard ratio (HR): 4.564, 95% CI: 0.583-35.7, *P* = .148). Liu et al^23^ found a non-significant impact on OS (HR: 1.285, 95% CI: 0.632-2.616, *P* = .489).

The results for SCCAg are not reliable enough for including the biomarker in routine practice to identify LN metastasis as the results are controversial, though there seems a tendency for higher levels in patients with LN metastases. The 2 studies that investigated the impact of SCCAg levels on patients’ survival reported non-promising results.

#### C-Reactive Protein

C-reactive protein (CRP) is synthesized by the liver in response to an inflammatory stimulus and was recorded in several conditions including infection, trauma, and malignant tumors.^25^ Elevated CRP levels in malignancies could be explained by the inflammatory state induced by tumor growth or the immune reaction triggered by tumor antigens.^26^ High plasma CRP levels were associated with poor prognosis in some cancers such as renal cell carcinoma.^27^

Steffens et al^28^ reported a significant association between high preoperative serum CRP levels above 15 mg/L and nodal disease (*P* = .007). In addition, patients with high CRP levels had a worse 5-year cancer-specific survival (CSS, *P* = .001). Multivariate analysis identified CRP as an independent prognostic factor for CSS (HR: 3.34, 95% CI: 1.04-10.72, *P* = .04). The study by Al Ghazal et al^29^ linked also high CRP serum levels above 20 mg/L with nodal metastasis. Li et al^19^ demonstrated that CRP levels ≥ 4.5 mg/L were significantly associated with extranodal extension (*P* < .001), pelvic LN metastases (*P* = .007), and 3-year CSS (*P* < .001) (Table 2). Multivariate regression analysis showed that the combined use of high CRP and SCCAg levels was significantly associated with 3-year CSS (HR: 3.39, 95% CI: 1.104-10.411; *P* = .033). Although the findings of the mentioned studies show promising results for the association of CRP with LN metastasis and patients’ survival, these results are too heterogeneous to accept CRP as a routine marker.

#### Ki-67

Ki-67 is a nuclear matrix protein that is considered a marker for cell proliferation. Eight studies^30-37^ assessed Ki-67 expression in PC patients with nodal metastasis. Three studies reported that Ki-67 expression was significantly associated with LN metastasis on univariate analysis.^33,34,37^ Moreover, 1 study even reported an inverse relationship, with low Ki-67 levels being associated with nodal disease.^31^ The other 4 studies^30,32,35,36^ reported the lack of significant association between Ki-67 levels and LN metastasis (Table 2).

The relationship between Ki-67 expression and survival rates was assessed in 6 studies.^32-36,38^ Two studies found that high Ki-67 levels significantly correlated with worse survival on univariate analysis,^33,34^ but multivariate analysis by May et al^33^ did not reveal a significant association. The remaining 4 studies^32,35,36,38^ did not find a significant association between elevated Ki-67 and reduced survival rates (Table 2).

The results concerning the use of Ki-67 as a marker for LN metastasis appear heterogeneous and thus the marker cannot currently be recommended to be used in clinical practice. As for the prediction of survival, the results indicate a poor impact on patients' survival, with no potential for clinical use.

#### Human Papillomavirus Status

Human papillomavirus (HPV) is a double-stranded DNA virus. Human papillomavirus is sexually transmitted and has been linked with PC, particularly the HPV 16 and 18 subtypes.^39,40^ The HPV infection binds—through the HPVE7 and E6 oncoproteins—to the host retinoblastoma and p53 proteins, disrupting apoptosis and leading to abnormal cellular proliferation.^41^ The HPV is detected in up to 50% of PC. Basaloid and warty PC show a high prevalence of HPV while verrucous and papillary PC exhibited HPV in approximately one-third of patients.^42^

As a marker for LN metastasis, 4 studies^43-46^ reported the lack of significant association between HPV positivity and nodal disease (Table 2).

Six studies evaluated the impact of HPV infection on survival in PC. Four studies stated the lack of significant association,^43,45-47^ while 2 studies^44,48^ reported that HPV infection was significantly associated with good outcomes and improved DSS (Table 2).

#### P16INK4a

P16INK4a is a tumor-suppressor gene that prevents cell division. Immunohistochemical staining in high-risk HPV genotypes demonstrated the overexpression of P16INK4a.^49^ Therefore, P16INK4a expression can be used as a surrogate biomarker for HPV infection.^50^ High sensitivity (100%) and relatively low specificity (57%) were reported for P16INK4a immunostaining when used as a predictor for high-risk HPV DNA.^51^

Positive P16INK4a immunoreaction was reported to have an inverse relationship with occult LN metastasis,^52^ but its performance as a predictor was poor and lacked statistical significance. Ferrándiz-Pulido et al.^46^ Tang et al.^53^ and Mohanty et al^54^ reported the lack of significant association with positive LNs (Table 2).

Positive P16INK4a immunoreaction has been associated with a tendency toward improved survival on univariate analysis in 4 studies^38,54-56^ and multivariate analysis in 3 studies.^38,55,56^ On the other hand, other studies showed a lack of significant association between P16INK4a immunoreaction and OS^46,53^ or CSS^51^ in univariate analysis. Steinestel et al^51^ stated that P16INK4a positivity and high-risk HPV status suggested a less aggressive behavior of PC, but no significant association was found with CSS. The multivariate analysis by Bethune et al^38^ showed that lacking p16 expression predicted worse OS (HR: 0.54, 95% CI: 0.31-0.93, *P* = .026), but not CSS (HR: 0.53, 95% CI: 0.26-1.06, *P* = .073; Table 2).

#### P53

TP53 is a tumor suppressor gene that exhibits mutations in approximately two-thirds of adult solid tumors.^57^ A mutated TP53 gene results either in producing an anomalous p53 protein (in 90% of cases) or its absence (in 10% of cases). The anomalous protein accumulates in the nucleus of cancer cells which can be demonstrated by immunohistochemical staining.^58^ The prevalence of p53 overexpression ranges from 26% to 91% in PC.^59,60^

Eight studies^23,36,54,56,59,61-63^ reported the relationship between p53 overexpression and positive LN in PC. Six studies found a significant association,^23,36,56,59,61,62^ with increased risk on univariate analysis ranging between 1.04 and 266.4. Multivariate analysis was conducted by Lopes et al.^59^ Zhu et al.^36^ and Liu et al^23^ who found p53 to be an independent predictor of LN metastasis, whereas Zhu et al^62^ found a non-significant association on multivariate regression (OR: 3.22, 95% CI: 0.96-10.86, *P* = .058; Table 2).

The relationship between p53 overexpression and survival was assessed by 8 studies.^36,38,54,56,59,61,63,64^ Five studies^36,59,61,63,64^ reported that overexpression of p53 was significantly associated with worse survival rates on univariate analysis. Multivariate analysis was performed in 4 studies,^23,36,61,63^ revealing a significant worsening of survival rates with positive p53. Three studies^38,54,56^ stated the lack of a significant relationship between p53 and survival (Table 2).

#### Programmed Death-Ligand 1

Programmed death-ligand 1 (PD-L1) is an immune-checkpoint marker whose expression was linked to advanced tumor stage and LN metastasis.^65^

Six studies^65-70^ assessed the relationship between PD-L1 expression in the primary tumor of the penis and the presence of LN metastasis. Two studies^66,70^ showed a significant association in univariate analyses between positive expression and LN metastasis, and another study^67^ showed a significant association when a diffuse pattern of expression was detected, compared to marginal expression. Multivariate analysis by Ottenhof et al^67^ found a significant impact of margin pattern compared to PD-L1 negative tumors (OR: 0.40, 95% CI: 0.16-0.99, *P* = .05). Hu et al^70^ reported also that positive PD-L1 was an independent predictor of LN metastasis (OR: 5.16, 95% CI: 1.29-20.58, *P* = .02). Hu et al^70^ developed a nomogram based on PD-L1 expression, tumor grade, lymphovascular invasion, and neutrophil-to-lymphocyte ratio to predict the preoperative risk of positive inguinal LNs in PC patients. On the other hand, no significant association with LN metastasis was detected in the 3 other studies,^65,68,69^ though they observed a trend for more frequent LN metastasis (Table 2).

Six studies^65-70^ evaluated the predictive value of PD-L1 for survival in PC patients. Significant association with CSS was reported by 3 studies^66, 68,70^ on univariate analysis. Moreover, multivariate analysis in 1 study^68^ showed a significant association with diffuse pattern (HR: 4.37, 95% CI: 1.04-18.32, *P* = .04). Meanwhile, Ottenhof et al^67^ found that positive PD-L1 expression of the tumor was not significantly associated with CSS at all tested cut-off values, but a diffuse PD-L1 expression predicted worse CSS in PD-L1+ tumors compared to tumors with marginal expression (HR: 3.92, 95% CI: 1.46-10.52, *P* = .01). Hu et al^70^ reported a significantly poorer 5-year CSS in PD-L1 positive patients (77.6% vs. 42%, *P* = .04) on univariate analysis by Kaplan–Meier curves, but the association was non-significant on multivariate analysis (HR: 1.58, 95% CI: 0.43-5.77, *P* = .49). The other 2 studies^65,69^ stated the lack of significant association (HR ranging from 1.65 to 2.13, *P* > .05) between PD-L1 and mortality/survival (Table 2).

#### Cytogenetics in Penile Cancer

Advances in genome studying techniques enable sequencing of the entire genome of a tumor cell, which opens the potential of identifying new biomarkers in cancer patients. Whole exome sequencing is used to identify genetic imbalance which is defined as “a genome showing any loss or gain of DNA sequences compared with the reference DNA whole sequence of the genome of interest.”^71^ The epigenetic analysis identifies potentially reversible alterations of the genome—such as methylation and histone modification—which are known as epigenetic modifications and may induce genetic instability.^72^

A genetic imbalance was reported in PC patients, as comparative genomic hybridization enabled the identification of DNA copy-number alterations.^73,74^ Copy-number alterations of 3p, 3q, and 8p were associated with reduced CSS and DSS. Several studies reported on the association between the amplification of the MYC gene with CSS in PC.^75,76^ The MYC gene is a proto-oncogene in the 8q24 chromosome that encodes a transcription factor responsible for regulating cellular proliferation, differentiation, and apoptosis.^77^ Busso-Lopes et al^74^ demonstrated the presence of MYC gene amplification in PC patients, but no prognostic significance was detected.

Another genetic imbalance in PC is the loss of heterozygosity on chromosomes 6, 9, and 12, which correlated with metastasis and advanced stage.^78^ These findings suggest a promising potential prognostic role of cytogenetic markers in PC patients.

There is a paucity of data on epigenetic modifications and their prognostic value in PC. One study^72^ identified methylation epi-signatures that were associated with HPV status and LN metastasis, with a sensitivity and specificity of 93% and 80%, respectively. Another study^79^ found an association of low brain-derived neurotrophic factor gene methylation with LN metastasis and a shorter DSS using univariate analysis, but this significance was not detected in multivariate analysis.

#### Current Ongoing Clinical Trials on the Management of Penile Cancer

The search continues to find new treatment lines for PC that can improve patient outcomes. Current clinical trials that are registered on Clinicaltrials.gov are listed in Table 3.

The sequencing of surgery and chemotherapy or radiotherapy is assessed in the AFU-GETUG 25 trial and the InPACT trial.

The AFU-GETUG 25 trial (NCT02817958) compares LN dissection (LND) and adjuvant chemotherapy to neoadjuvant chemotherapy followed by bilateral LND. The chemotherapy regimen (TIP) includes paclitaxel, ifosfamide, and cisplatin for 4 cycles every 3 weeks. The estimated study completion date is in September 2024. This non-randomized, open-label trial targets the enrolment of 37 participants. The first arm will be subjected to adjuvant chemotherapy TIP after modified bilateral LND (4 cycles every 21 days). The second arm will undergo fine needle biopsy or sentinel node + neoadjuvant chemotherapy TIP followed by modified bilateral LND. The primary outcome is survival without locoregional LN recurrence. The eligibility criteria included adult men with histologically proven PC, stage cN1 and cN2 or nodes involvement risk ≥ pT1b and/or grade 2. The secondary outcomes include a complete response rate to neoadjuvant chemotherapy, survival, toxicity, and quality of life.

The InPACT trial (NCT02305654) is a phase III, open-label, randomized trial that assesses the sequencing of surgery, chemotherapy, and chemoradiotherapy. The study is still recruiting, and the estimated sample size is 400 participants. The study enrolls adult men with histologically proven SCC of the penis, any T stage, N1 to N3 nodal stages, and no metastasis. The primary outcome is OS, while secondary outcomes include DSS, grade 3 or 4 toxicity, disease-free survival, surgical complication, quality of life, and pathological complete remission.

Several studies are evaluating the use of new drugs in PC patients. The ORPHEUS phase II trial (NCT04231981) evaluates INCMGA00012, a new drug acting on PI3K and indoleamine 2,3-dioxygenase in patients with advanced stages. The study recruited 18 patients and its completion date is estimated to be in December 2022. Eligible patients are those above 18 years old, with histologically proven penile SCC, locally advanced unresectable or metastatic stage 4 cancer. The primary outcome is the objective response rate, and the secondary outcomes include clinical benefit rate, progression-free survival (PFS), duration of response, OS, maximum tumor shrinkage, and adverse events.

Several clinical trials are assessing anti-PD-L1 monotherapy in PC patients. Three clinical trials are evaluating avelumab.

The open-label, single-arm, phase II ALPACA trial (NCT03391479) assesses avelumab in patients with advanced PC (locally advanced or metastatic) who are either unfit for or progressing on platinum-based chemotherapy. The study aims to enroll 24 patients. The studied outcomes include objective response rate (primary) as well as PFS and OS (secondary outcomes). Patients are eligible for enrolment if they are above 18 years, with histologically proven SCC of the penis, unresectable/metastatic stage, and unfit for platinum-based chemotherapy or progressed on/after treatment with platinum-based chemotherapy.

The PULSE trial (open-label, single-arm, NCT03774901) uses avelumab as maintenance therapy in stable diseases with first-line chemotherapy and will enroll 32 participants. The eligibility criteria include age above 18 years and histologically confirmed unresectable locally advanced or metastatic penile SCC. The studied outcomes are PFS (primary) as well as OS, quality of life, and adverse events (secondary outcomes).

The LATENT study (NCT03357757, open-label, single-arm) evaluates the combined use of avelumab and valproic acid in SCC patients with advanced p16-positive tumors. The study estimates to enroll 39 participants. The study will include patients above 18 years old of either sex and with confirmed SCC of the penis, cervix, vulva, vagina, or anus. The primary outcomes are the efficacy of the intervention and the proportion of patients completing 4 doses of the treatment. Secondary outcomes are OS, PFS, adverse events, and immunoscore.

Atezolizumab is under evaluation in an open-label, phase-II study (PERICLES; NCT03686332) with or without radiotherapy in advanced penile SCC. The study enrolled 32 men ≥18 years of age, with advanced histologically documented SCC of the penis or distal urethra. The primary endpoint is PFS, and the secondary endpoints are OS and the percentage of patients completing the full course of radiotherapy.

Pembrolizumab is another drug under assessment in 2 trials. An open-label, single-arm, phase-II trial (HERCULES; NCT04224740) assesses the drug in advanced PC along with the standard-of-care chemotherapy. The study targets to enroll 33 adult men with penile SCC and metastatic disease or recurrent locally advanced disease not amenable to curative therapy. The primary outcome is the overall response rate. The secondary outcomes are PFS, OS, clinical benefit rate, and quality of life. The open-label, single-arm PEVOsq trial (NCT04357873) uses pembrolizumab in association with vorinostat in 111 patients with recurrent and/or metastatic SCC of different body regions. Patients must have histologically confirmed recurrent and/or metastatic SCC of the head and neck, cervix, lung, anus, vulva, or penis and radiologically confirmed progressive recurrent and/or metastatic disease. The studied outcomes include the objective response rate (primary) as well as the duration of response, PFS, and OS.

The human anti-PD-L1 monoclonal antibody is being assessed in an open-label single-arm, phase II trial (NCT04718584) enrolling 127 patients with tumors of the urinary and genital systems (muscular-infiltrating bladder cancer suitable for surgery, advanced opaque cell renal carcinoma, and advanced PC). The primary outcome is a complete response, whereas the secondary outcomes are recurrence-free survival, PFS, disease control rate, duration of response, OS, and adverse events.

The role of gene-modified HPV virus (MEDI0457) is evaluated in an open-label, single-arm, phase II clinical trial (NCT03439085), along with durvalumab for recurrent or metastatic HPV-related cancers, including PC. The study enrolls 77 adult participants of either sex with recurrent/metastatic HPV-associated cancers and cancers refractory to standard therapy. The primary outcome is the objective response rate. The secondary outcomes comprise the disease control rate, PFS, and OS.

## Conclusion

New tumor biomarkers may allow for tailoring personalized therapy in cancer patients by identifying those at high risk of LN metastasis who will benefit from pelvic LND, radiotherapy, chemotherapy, or combination treatments. Several biomarkers for PC have been identified, but heterogeneity in outcomes and no improvement beyond current normal practice means the non-feasibility of incorporating these biomarkers in patient management currently. The lack of standard definitions of the markers’ positivity or the optimal cut-off values may contribute to this. In addition, studies were not powered for conducting regression analysis to adjust for confounders due to their relatively small sample sizes.

Further collaborative research is necessary to validate the incorporation of current and new biomarkers in the management of PC. The new therapeutic agents being investigated, such as checkpoint inhibitors, may enhance the response and therefore reduce the administered dose of cytotoxic drugs or radiation which will decrease the adverse effects of therapy or may allow treatment to be directed toward those who will benefit most.

## Figures and Tables

**Table 1.** Survival Rates in Penile Cancer Patients According to the Stage of Nodal Involvement

N Stage		3-Year Recurrence-Free Survival	5-Year Recurrence-Free Survival	5-Year Overall Survival
N0	No regional LN metastasis		93.4%^11^	56.2%^10^
N1	Metastasis in a single inguinal LN	90.6%^9^	89.7%^11^	49.0%^10^
		69.8%^12^		
N2	Metastasis in multiple or bilateral inguinal LNs	64.1%^9^	30.9%^11^	67.6%^10^
		48.2%^12^		
N3	ENE of LNM or pelvic LN(s), unilateral or bilateral	15.5%^9^	0%^11^	19.4%^10^
		33.3%^12^		

ENE, extranodal extension; LN, lymph node; LNM, lymph node metastasis.

**Table 2.** Summary of the Reported Biomarkers’ Association with Lymph Node Metastasis and Survival in Penile Cancer Patients

Biomarker	LN Metastasis	Survival
SCCAg	Significant association of high levels with LNM on univariate^19,22^ and multivariate analysis.^18,20^ No significant association on univariate analysis.^21,23,24^	Non-significant impact in multivariate analysis on OS (HR: 1.285, 95% CI: 0.632-2.616, *P* = .489)^23^ or DSS (HR: 4.564, 95% CI: 0.583-35.7, *P* = .148).^19^.
CRP	Significant association of high levels with LNM on univariate analysis.^19,28,29^	Significant association of high CRP level with lower CSS rate on univariate^19,28^ and multivariate analysis (CRP > 15 mg/L, HR: 3.34, 95% CI: 1.04-10.72, *P* = .04).^28^
Ki-67	Significant association on univariate^31,33,34,37^ and multivariate analysis (RR: 3.73; 95% CI 1.4-9.7, *P* = .01) ^31^ Non-significant association on univariate analysis.^32,35^	Significant association on univariate analysis.^33,34^ No significant association on univariate^32,35,36,38^ or multivariate analysis.^33,38^
HPV	Non-significant association on univariate analysis.^43-46^	Significantly shorter DSS in the high-risk HPV-negative group on univariate and multivariate analysis (HR: 0.14-0.2, *P* < .05).^44, 48^ No significant impact in univariate analysis on OS^43,45-47^ or CSS.^45^ Likewise on OS in multivariate analysis.^47^
P16INK4a	Significant association on univariate analysis of negative p16 with LNM.^52^ No significant association on univariate analysis.^46,53,54^	Significant association of negative expression with improved OS on univariate.^38,54,56^ and multivariate analysis (HR: 0.54, 95% CI: 0.31-0.93, *P* = .026) ^38^ Significant association of negative expression with improved CSS on univariate analysis^54, 55^ and multivariate analysis (HR: 0.36-0.44, *P* < .05) ^55, 56^ No significant impact on OS in univariate analysis^46, 53^ and CSS in univariate analysis^51^ or multivariate analysis (HR: 0.53, 95% CI: 0.26-1.06, *P* = .073) ^38^
TP53	Significant association on univariate analysis of positive p53 with LNM^23,36,56,59,61,62^ and multivariate analysis (OR: 6.01-22.431, *P* < .01).^23,36,59^ No significant association on univariate analysis^63^ or multivariate analysis (OR: 3.22, 95% CI: 0.96-10.86, *P* = .058) ^62^	Negative p53 had a significantly better OS on univariate analysis^59, 64^ and multivariate analysis (OR: 5.997, 95% CI: 1.615-22.275).^23^ Significantly better CSS on univariate analysis^36,61,63^ and multivariate analysis.^36,61,63^ No significant effect in univariate analysis on OS^38,54^ or CSS.^38,54,56^
Programmed death-ligand 1	Significant association on univariate analysis^66,67,70^ and multivariate analysis (Margin vs. PD-L1- OR: 0.40, 95% CI: 0.16-0.99, *P* = .05^67^ and OR: 5.16, 95% CI: 1.29-20.58, *P* = .02).^70^ No significant association on univariate analysis.^65,68,69^	Significant association on univariate analysis with decreased CSS (*P* = .011)^66,68,70^ and multivariate analysis (HR: 4.37, 95% CI: 1.04-18.32, *P* = .04) ^68^ and DSS.^67^ No significant effect in univariate analysis on OS^69^ or CSS in multivariate analysis (1.58-1.65, *P* < .05).^65,70^

CSS, cancer-specific survival; DSS, disease-specific survival; HR, hazard ratio; LN, lymph node; LNM, lymph node metastasis; OR, odds ratio; OS, overall survival.

**Table 3.** Ongoing Studies Investigating New Treatment Lines for Penile Cancer (from clinicaltrials.gov)

NCT Number	Acronym	Interventions and Arms	Phases	Start Date	Completion Date	Last Update	Status
NCT02817958	AFU-GETUG 25	Arm A: adjuvant chemotherapy TIP (Paclitaxel, ifosfamide, & cisplatin) after modified bilateral lymphadenectomy Arm B: fine needle biopsy or sentinel node + neoadjuvant chemotherapy TIP followed by a modified bilateral lymphadenectomy	Phase II	October 17, 2016	September 2024	September 28, 2021	Recruiting
NCT02305654	InPACT	Arm A: ILND Arm B: neoadjuvant chemotherapy followed by ILND Arm: C. neoadjuvant chemoradiotherapy followed by ILND Arm P: prophylactic PLND Arm Q: Surveillance no prophylactic PLND	Phase III	May 12, 2017	July 2022	October 31, 2019	Recruiting
NCT04231981	ORPHEUS	Single arm: INCMGA00012 500 mg on day 1 of each cycle (once every 4 weeks), for up to 2 years.	Phase II	April 28, 2020	December 2022	June 3, 2022	Active, not recruiting
NCT03391479	ALPACA	Single arm: Avelumab 10 mg/kg IV, once every 2 weeks	Phase II	August 15, 2018	June 30, 2023	December 9, 2021	Recruiting
NCT03774901	PULSE	Single arm: Avelumab 10 mg/kg IV every 2 weeks	Phase II	March 12, 2019	September 22, 2024	February 24, 2022	Recruiting
NCT03357757	LATENT	Single arm: Valproic acid (12.5 mg/kg) once per day and Avelumab (10 mg/kg IV) every 2 weeks for up to 2 years	Phase II	February 7, 2018	February 26, 2027	July 5, 2019	Recruiting
NCT03686332	PERICLES	Arm A: Atezolizumab and radiotherapy Arm B: Atezolizumab	Phase II	September 25, 2018	December 1, 2023	April 15, 2022	Active, not recruiting
NCT04224740	HERCULES	Arm A: Pembrolizumab 200 mg IV once every 3 weeks maximally for 2 years Arm B: Cisplatin plus 5-Fluorouracil for 6 cycles	Phase II	June 15, 2020	December 2025	February 18, 2022	Recruiting
NCT04357873	PEVOsq	Single arm: Pembrolizumab: 200 mg every 3 weeks, up to 35 administrations Vorinostat: 400 mg once daily, till progression	Phase II	October 28, 2020	October 2024	June 18, 2021	Recruiting
NCT04718584		Single arm: LDP 10 mg/kg once every 2 weeks. Surgical treatment within 2 weeks after the end of 3 cycles of treatment.	Phase II	September 11, 2020	November 2023	January 22, 2021	Recruiting
NCT03439085		Single arm: MEDI0457 & Durvalumab	Phase II	November 14, 2018	December 31, 2022	February 14, 2022	Active, not recruiting

HPV, human papillomavirus; ILND, inguinal lymph node dissection; IV, intravenous; PLND, pelvic lymph node dissection.
